# Supplementary material for: Development, evaluation and application of a novel markerless motion analysis system to understand push-start technique in elite skeleton athletes
Source: PLoS One. 2021 Nov 15;16(11):e0259624. doi: 10.1371/journal.pone.0259624 (PMC8592484; doi:10.1371/journal.pone.0259624)
Supplement: S1 File — (PDF) [file pone.0259624.s001.pdf]

Supporting information

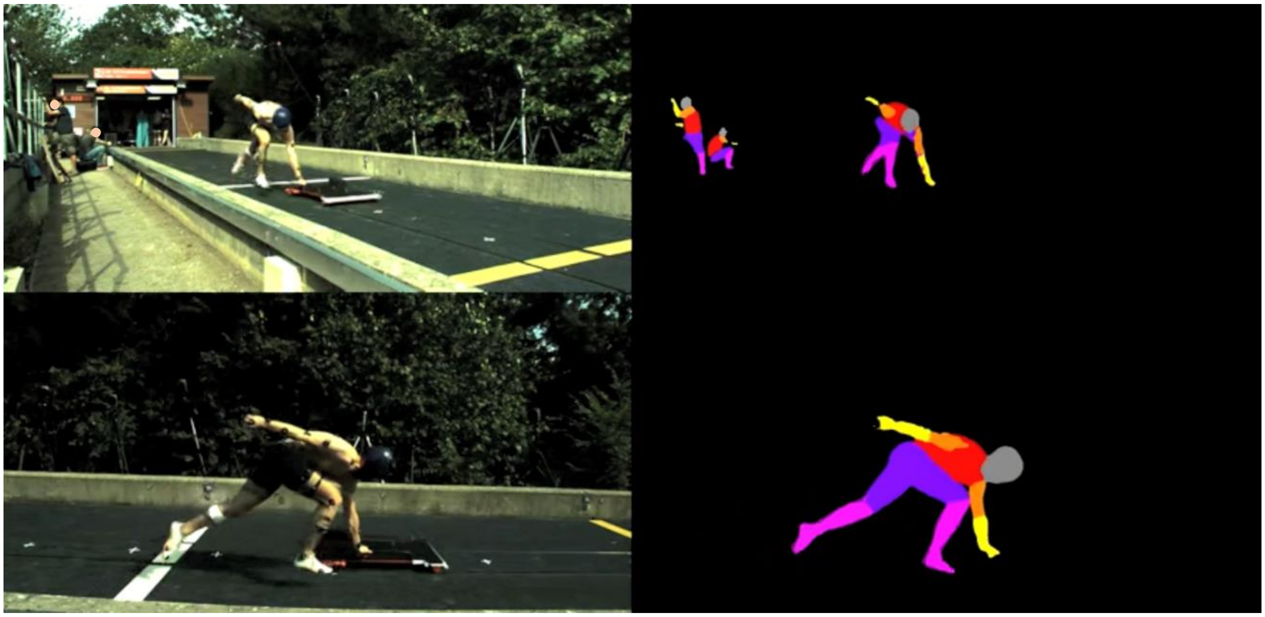

Figure S1. *Example image segmentation from the CDCL-human-part-segmentation network. Left images depict raw images from two camera fields of view. Right images depict each detected person segmented with a foreground mask.*

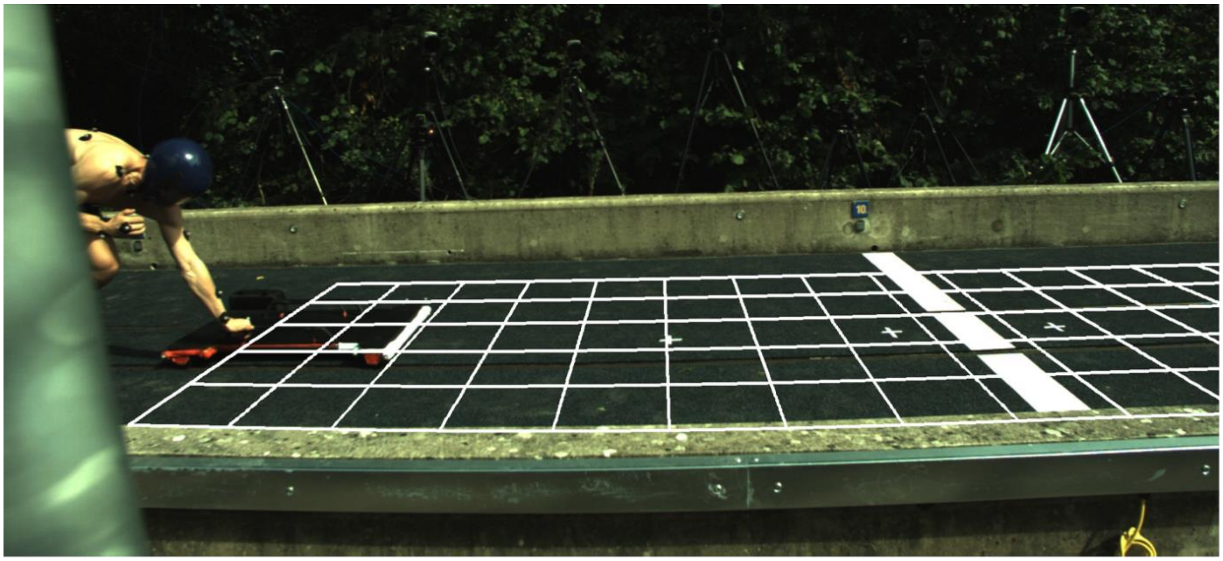

Figure S2. *Example floor plane grid projected onto a view of the track. This plane was raised 2.5 cm to reduce noise and occlusion caused by the sled and was used to detect ground contact events by assessing the occupancy of each grid during each time instance.*

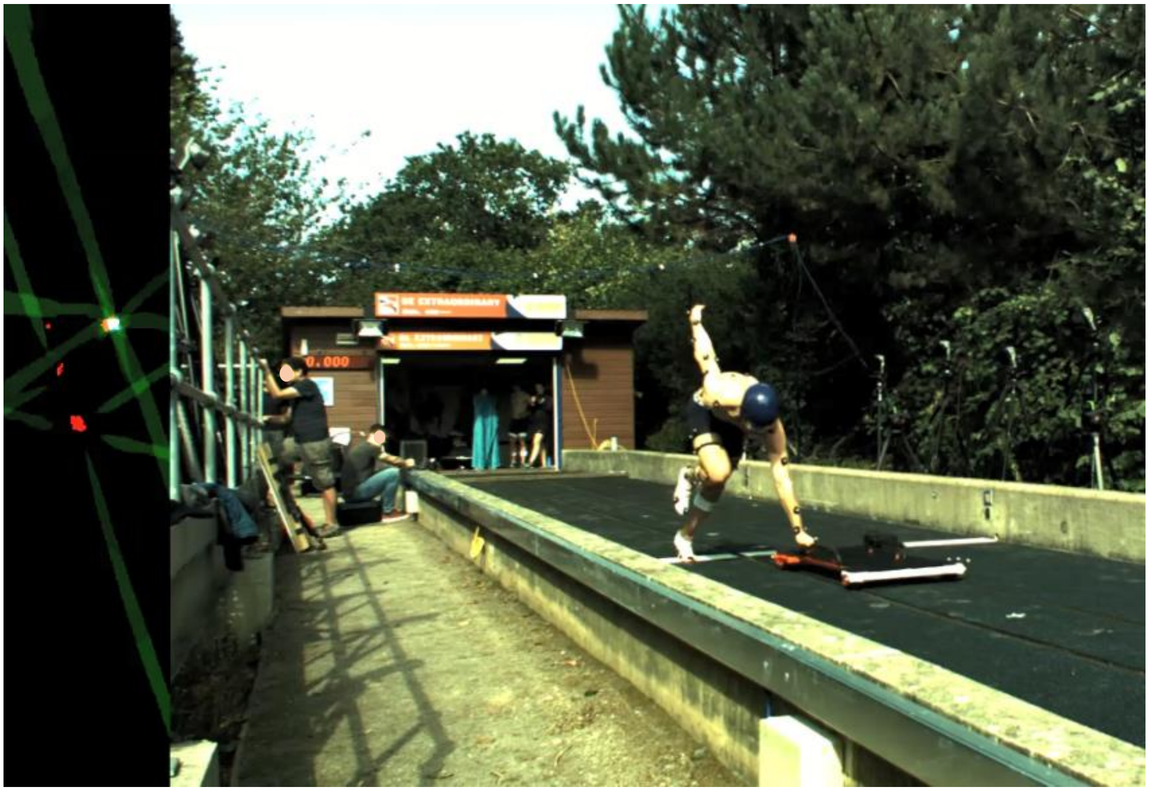

Figure S3. *An example ground contact shown in the main image is detected by assessing the activity of the occupancy map, which is shown on the left side or this image. The dark strip represents the ground plane of the track with the athlete travel from top to bottom. Brighter cells indicate that a greater number of cameras are contributing to the additive project of the foot in contact with the ground plane.*

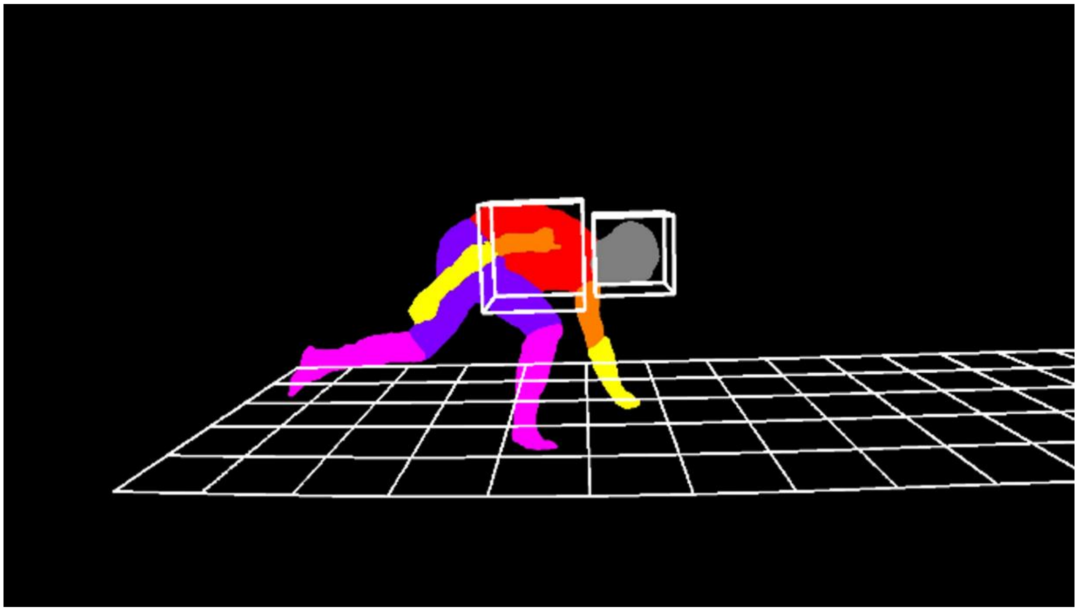

Figure S4. *The optimised 3D bounding boxes for the torso and head.*

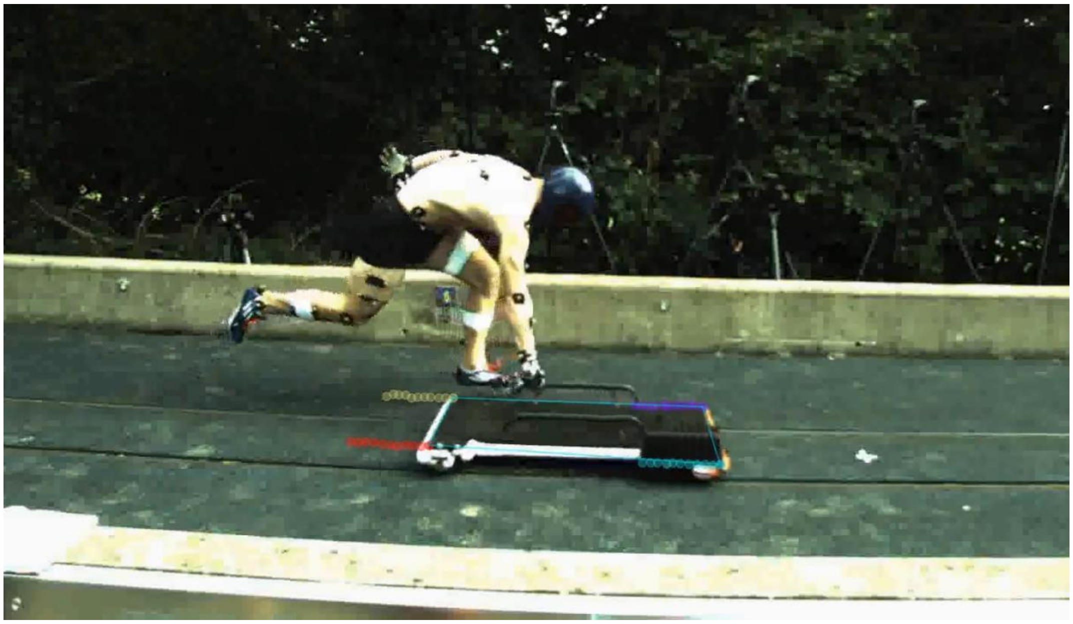

Figure S5. *Examples of sled corner detections. A detection history of 10 frames is depicted by the trail of coloured markers on each sled corner.*

Table S1: Estimation statistic results showing step characteristic differences (asymmetries) for both pushing and sprinting.

Variable	Paired Mean Difference	Paired Cohen's *d*	CI Lower Bound	CI Upper Bound	*P* Value
SL - Push	-0.22	-2.89	-3.55	-2.16	< 0.001
SL - Sprint	-0.01	-0.02	-0.12	0.07	0.643
SF - Push	0.33	1.54	0.89	2.20	< 0.001
SF - Sprint	0.03	0.14	-0.09	0.39	0.214
SV - Push	-0.36	-1.02	-1.44	-0.63	< 0.001
SV - Sprint	0.03	0.02	-0.07	0.10	0.632
GCT - Push	-0.01	-0.07	-0.52	0.30	0.701
GCT - Sprint	-0.01	-0.04	-0.12	0.04	0.298
FT – Push	-0.02	-1.88	-2.64	-1.21	< 0.001
FT - Sprint	-0.00	0.01	-0.13	0.13	0.980

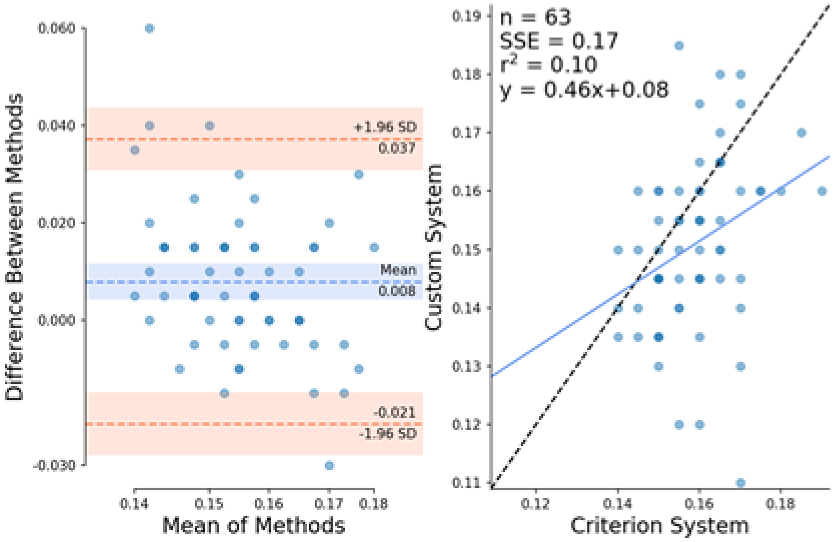

Figure S6. *Bland-Altman and linear regression plots comparing ground contact times between systems. Confidence intervals are given around the mean difference and 95% limits of agreement.*

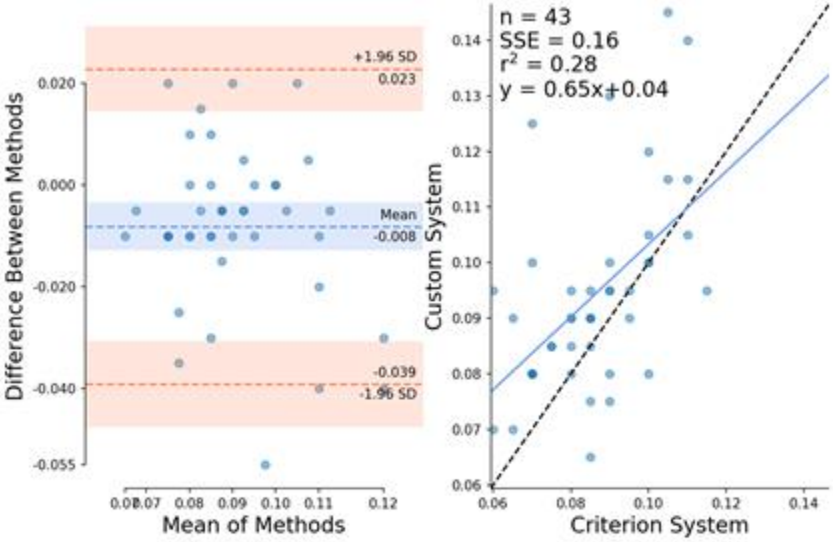

Figure S7. *Bland-Altman and linear regression plots comparing flight times between systems. Confidence intervals are given around the mean difference and 95% limits of agreement.*

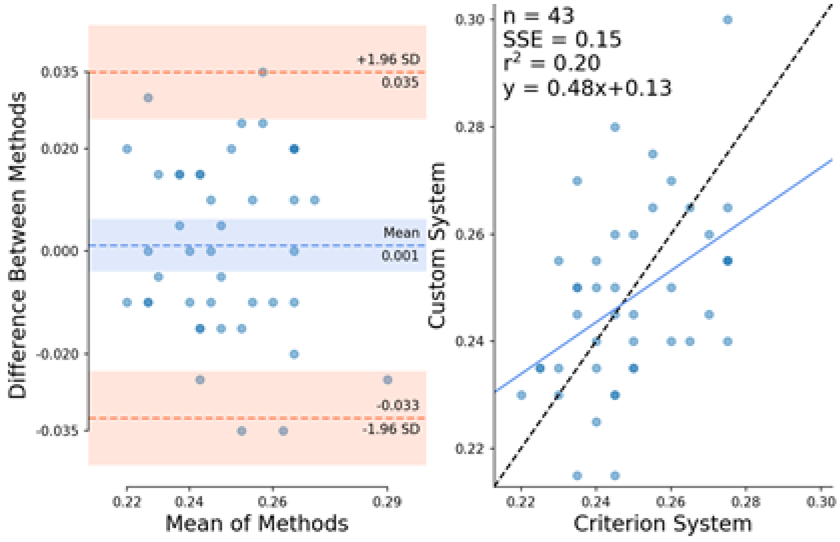

Figure S8. *Bland-Altman and linear regression plots comparing step times between systems. Confidence intervals are given around the mean difference and 95% limits of agreement.*

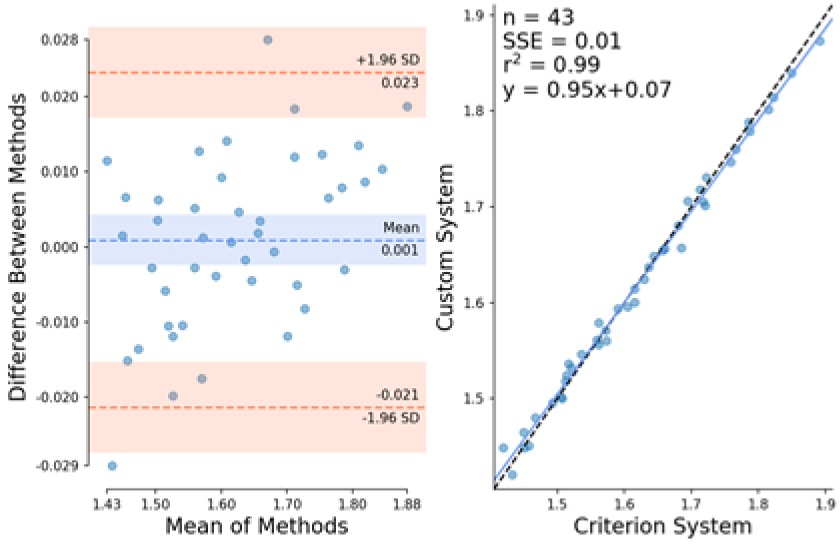

Figure S9. *Bland-Altman and linear regression plots comparing step lengths between systems. Confidence intervals are given around the mean difference and 95% limits of agreement.*

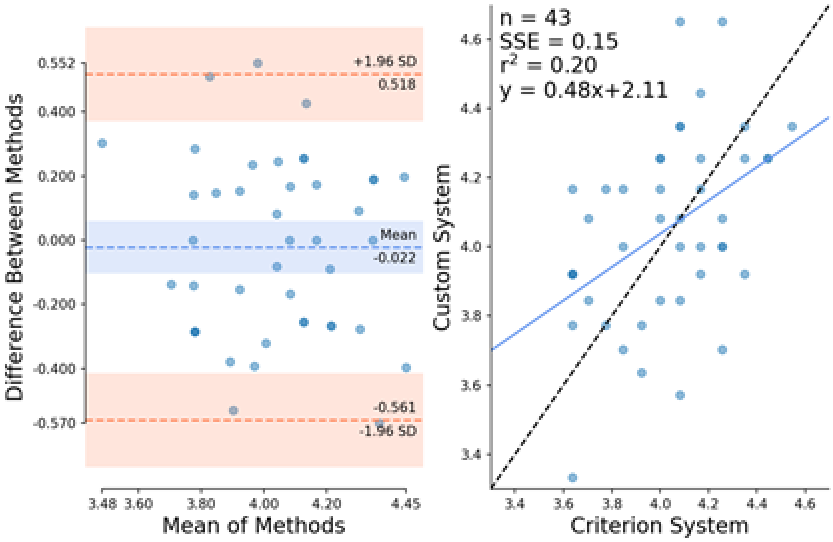

Figure S10. *Bland-Altman and linear regression plots comparing step frequency between systems. Confidence intervals are given around the mean difference and 95% limits of agreement.*

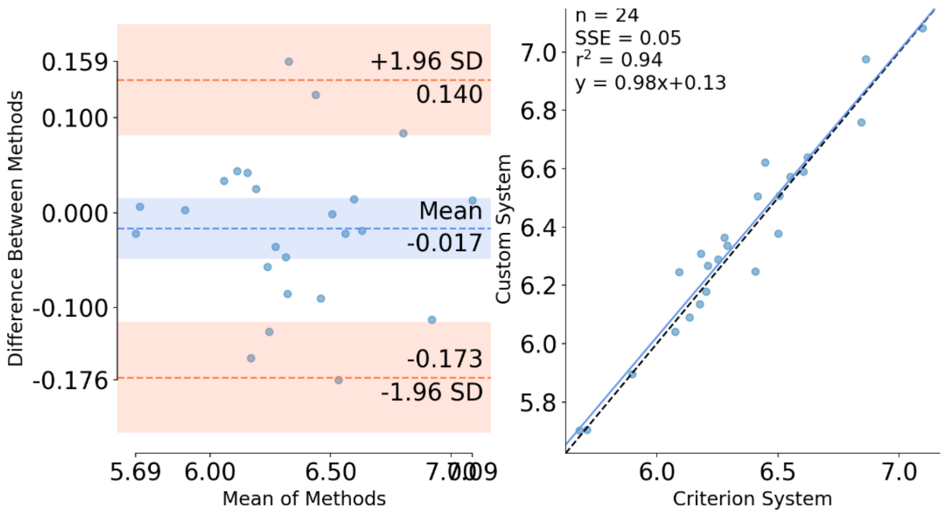

Figure S11. *Bland-Altman and linear regression plots comparing step averaged athlete centre of mass velocity during pushing between systems using the 3D bounding box method. Confidence intervals are given around the mean difference and 95% limits of agreement.*

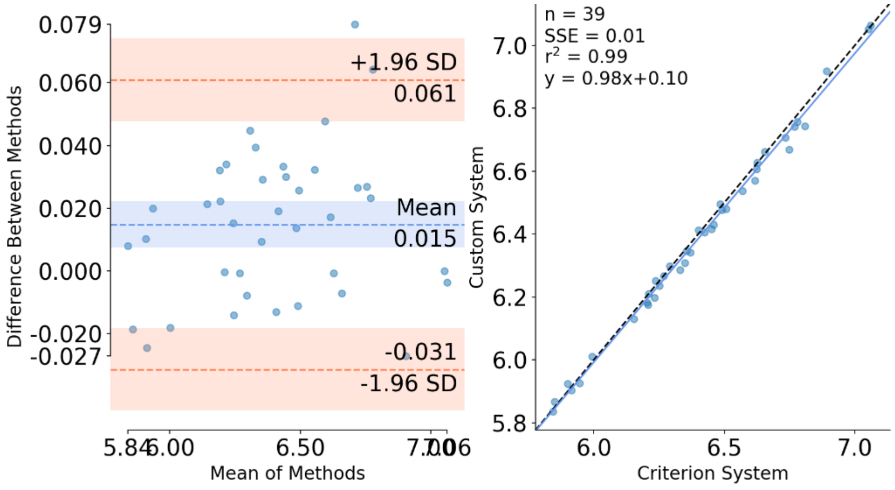

Figure A12. *Bland-Altman and linear regression plots comparing step averaged sled centre of mass velocity between systems using the 3D bounding box method. Confidence intervals are given around the mean difference and 95% limits of agreement.*

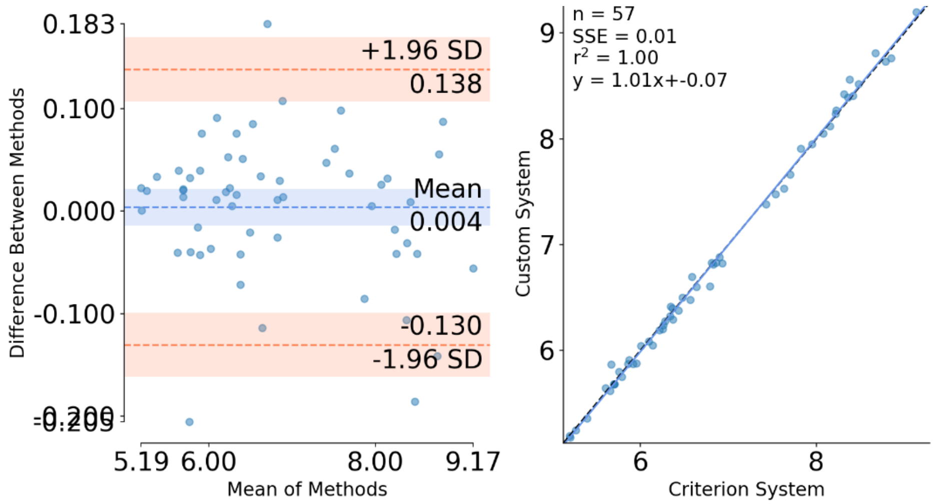

Figure A13. *Bland-Altman and linear regression plots comparing step averaged athlete centre of mass velocity during sprinting between systems using the 3D bounding box method. Confidence intervals are given around the mean difference and 95% limits of agreement.*
