# Supplementary material for: Development, evaluation and application of a novel markerless motion analysis system to understand push-start technique in elite skeleton athletes
Source: PLoS One. 2021 Nov 15;16(11):e0259624. doi: 10.1371/journal.pone.0259624 (PMC8592484; doi:10.1371/journal.pone.0259624)
Supplement: Attachment — Submitted filename: Response_to_Reviewers_rev2.pdf [file pone.0259624.s002.pdf]

Response to Reviewers – Round 2

Reviewer #2:

I have read the revised paper and the response to the reviewers. It is a good project report but not a scientific research paper.

We respectfully disagree with this opinion. In addition to the novel scientific contributions which we outlined in our previous response, the paper in question demonstrates that computer vision based technologies can be applied to have real-world impact. This potential impact could fall across a range of science and medicine disciplines, beyond the specific application (or 'project') of skeleton studied here. For example, the principles demonstrated in our paper could be applied by motor rehabilitation scientists and clinicians who quantify movement to inform rehabilitation design and evaluate the effects of disease and treatments. Neuroscientists studying brain-movement interaction and motor learning. Similarly, psychologists could examine human motor development, human motor behavior and the effects of psychological disorders on movement. Sports and exercise physiologists and biomechanists could examine the metabolic costs of human movement, sports techniques (as in our paper), injury mechanisms and equipment design. Finally, engineers could quantify movement for prosthetics, exoskeleton, and rehabilitation robotics design. The scope and breadth of these examples demonstrate the substantial impact that using computer vision technologies applied to human movement research can makes to science and medicine. As such, we feel that we have provided a clear and justified rationale for the scientific contribution this paper makes and why it is suitable for publication in PLOS One.

Reviewer #3:

The authors have well addressed all my comments except 2, that remained not understood by the authors but also maybe because of the lack of clarity of these 2 comments.

The 2 comments are actually linked.

Comment 1: "The authors also present first motion analysis results which i found a bit confusing since the accuracy of the system {i precise today i was talking about the markerless system] for such dynamic action still needs to be established"

Comment 2: "It is interesting that the authors develop on some findings on their trial and new system. But since it is a novel system, it would be appreciable to see if these results could be verified on the ground-truth system."

My concern with these 2 comments is that biomechanical interpretations are given thanks to the results of the markerless system. While the validity of the system has been quantified previously against a marker-based system, would the conclusions drawn by the authors would have been exactly the same if the data coming from the marker-based system were used instead? The assymetries that are depicted are that clear that i presume it is the case but if so, the authors could mention it.

Thank you for clarifying these specific comments. For clarification, any variable that has been presented in this study using our markerless system, has been verified against the ground-truth data (marker-based motion capture). Any systematic or random differences that were reported between the two measurement systems were shown to be small enough to allow for meaningful changes in push start and sprinting technique to be detected.

Differences between the systems and thus the accuracy of our markerless system, in relation to the ground truth (marker-based motion capture) are presented in Tables 1 and 2 as well as in the Supporting Information.

I have a last question. Why comparison results for GCT, FT etc are given only for pushing and not on sprinting? These variables are analyzed in fig 3 and 4 but we do not know their accuracy.

As is discussed in the methods section, sprinting validation results for GCT, FT etc are presented in our groups previous work using an earlier iteration of the methods presented in this study. For full details see Evans M, Colyer S, Cosker D, Salo A, Ieee, editors. Foot Contact Timings and Step Length for Sprint Training. 18th IEEE Winter Conference on Applications of Computer Vision (WACV); 2018 Mar 12-15; Nv2018.
